# Cardiac troponin patterns in athletes completing Norseman Xtreme Triathlon

**DOI:** 10.1136/bmjsem-2025-002810

**Published:** 2026-05-17

**Authors:** Martin Bonnevie-Svendsen, Erle Viktoria Edvardsson, Kjersti Oppen, Jørgen Melau, Christoffer Nyborg, Frode Wirum Sæter, Kristian Berge, Torbjørn Omland, Jonny Hisdal

**Affiliations:** 1 Clinical Medicine, University of Oslo Faculty of Medicine, Oslo, Norway; 2 Vascular Surgery, Section of Vascular Investigations, Oslo University Hospital Aker Hospital, Oslo, Norway; 3 KG Jebsen Centre for Cardiac Biomarkers, University of Oslo Institute for Clinical Medicine, Oslo, Oslo, Norway; 4 Laboratory Medicine, Vestre Viken Hospital Trust, Drammen, Buskerud, Norway; 5 Norwegian Armed Forces Joint Medical Services, Sessvollmoen, Norway; 6 Cardiology, Akershus University Hospital, Lørenskog, Akershus, Norway

**Keywords:** Cardiovascular, Cardiology, Physical activity, Triathlon, Biochemistry

## Abstract

**Objective:**

Cardiac troponins (cTns) are frequently measured in patients when suspecting coronary artery disease (CAD). However, recent studies suggest that elevated cTns following exercise may predict CAD and increased mortality. Although the mechanisms underlying postexercise cTn increase remain unclear, the cardiac troponin I (cTnI)/cardiac troponin T (cTnT) ratio shows promise for distinguishing cardiac pathologies. This study assesses the dynamics of cTnI and cTnT concentrations and the cTnI/cTnT ratio in serum samples collected 1–2 days before, within 1 hour after and 11–24 hour after completing Norseman Xtreme Triathlon.

**Methods:**

Using Abbott’s STAT High Sensitive Troponin-I assay and Roche’s Elecsys Troponin T hs assay, we measured cTnI and cTnT in venous serum of 93 adult triathletes.

**Results:**

Concentrations of cTnI, cTnT and cTnI/cTnT ratio increased from baseline to immediately after the race (cTnI median (25–75 percentile) from (3 (2–6) ng/L) to 69 (37–156) ng/L, cTnT from (6 (5–8) ng/L) to 40 (24–55) ng/L and cTnI/cTnT from (0.5 (0.3–0.8)) to 2.1 (1.5–2.9), all p<0.001). Serum cTnI and cTnT concentrations remained elevated the day after the race, whereas the cTnI/cTnT ratio declined the day after the race compared with baseline levels. Three participants displayed higher cTnI levels, and 14 participants showed higher cTnI/cTnT ratios the day after the race compared with immediately after.

**Conclusion:**

Serum concentrations of cTnI, cTnT and the cTnI/cTnT ratio increase immediately after a long-distance triathlon, and concentrations trend towards normalisation the next day. The clinical implications are unknown and warrant further investigation.

**WHAT IS ALREADY KNOWN ON THIS TOPIC**

- Transient elevated cardiac troponin (cTn) concentrations are commonly described after endurance training and may predict cardiovascular disease (CVD). However, the clinical interpretation remains unclear.

**WHAT THIS STUDY ADDS**

- Both cTn and the cardiac troponin I (cTnI)/cardiac troponin T (cTnT) ratio increased immediately after a long-distance triathlon. In addition, some individuals, including one with known CVD, demonstrated prolonged cTn elevation and increasing cTnI/cTnT ratios.

**HOW THIS STUDY MIGHT AFFECT RESEARCH, PRACTICE OR POLICY**

- The cTnI/cTnT ratio may be useful for predicting CVD in endurance athletes.

## Introduction

 Circulating levels of cardiac troponin (cTn) are often elevated after exercise without symptoms or signs of myocardial injury. While traditionally viewed as benign, elevated cTn above the 99th percentile upper reference limit (URL) in a healthy population shortly after exercise has been associated with a doubling of the risk (HR 2.48) of death and future cardiovascular events in middle-aged individuals.^1^ Exercise-induced cTn release has also been linked to signs of myocardial fibrosis,^2^ while other studies have failed to replicate this observation.^3 4^ Finally, recreational cyclists with obstructive coronary artery disease (CAD) display greater cTn concentrations 24 hours after racing compared with cyclists without CAD (mean cTnI 151 ng/L vs 24 ng/L, mean cTnT 39 ng/L vs 20 ng/L).^5^

The mechanisms behind exercise-induced cTn elevations are poorly understood and lack clinical confirmation.^1 6 7^ Interestingly, experimental data suggest that cardiac troponin I (cTnI) is cleaved and released faster than cardiac troponin T (cTnT) during myocyte injury.^8^ Indeed, while cTnI and cTnT correlate closely after myocardial infarction (MI) (r=0.78),^9^ MI is associated with cTnI concentrations up to 10-fold that of cTnT.^10^ However, association with chronic kidney disease is stronger for cTnT compared with cTnI.^11 12^ Furthermore, the ratio of high-sensitivity cTnI to high-sensitivity cTnT has shown good discrimination between type 1 and type 2 MI (area under the receiver-operator characteristic curve 0.81, 95% CI 0.74 to 0.87)^9^ and is positively associated with cardiovascular death in the general population, while inversely associated with non-cardiovascular death.^13^ However, the prognostic and clinical relevance of the cTnI/cTnT ratio after exercise remains unexplored, and the current study may therefore be a first step in elucidating the implications of the cTnI/cTnT ratio after prolonged endurance exercise.

Work duration and intensity appear to be important factors influencing the cTn response after exercise.^14^ The Norseman Xtreme Triathlon (NXTRI) is a triathlon race with a course distance of 225 km and a total elevation gain of more than 5000 m. The high workloads make this event an interesting venue for exploring the physiology and potential pathophysiological mechanisms involved in cTn release following severe endurance exercise. This prospective explorative study aimed to describe serum concentrations of cTnI, cTnT and the cTnI/cTnT ratio in adult triathletes before, immediately after and the day after a long-distance triathlon event.

## Methods

### Design and study population

Adult triathletes participating in the NXTRI were recruited in 2018, 2021, 2022, 2023 and 2024. Invitations for voluntary participation were posted on the organiser’s website a month before each event. Due to the explorative nature of this study, the only inclusion criterion was age 18 years or older. All participants were provided with written and verbal information about the study and signed informed consent. On enrolment, a questionnaire was issued to all participants to collect past medical history, use of medication and use of performance-enhancing drugs. The study was approved by the Regional Committee for Medical and Health Research Ethics in Norway (REK Sør-Øst 2016/932, 481115 and 621976) and conducted according to the Declaration of Helsinki.

### Race characteristics

The NXTRI race consists of 3.8 km swimming, 180 km cycling and 42 km running. The fastest 160 athletes finish at Mount Gaustatoppen at 1880 m altitude and remaining finishers complete the race at a second goal line at 1100 m altitude. The race is characterised by water temperatures as low as 14°C and ambient temperatures down to 6°C.^15^

### Blood sampling and laboratory analyses

Blood samples were collected from the antecubital vein within 40 hours before the races, within 1 hour after completing the race and 11- to 24-hour (mean±SD 15.4±1.9 hour) after the race into vacutainers containing K_2_ ethylenediaminetetraacetic acid and silica particles and gel separators. Whole blood samples were refrigerated 30–60 min after sampling while serum samples were allowed to clot at room temperature for 30 min before centrifuging at 2000×*g* for 10 min. Serum was then pipetted into freeze-resistant vials and refrigerated on transport to Oslo University Hospital (Aker) where vials were stored at −80°C until analysis of cTnI in October 2024. Each year, a batch of whole blood and serum was transported to a private certified clinical laboratory (Fürst medisinsk laboratorium, Oslo, Norway) and analysed for haemoglobin, N-terminal pro-B-type natriuretic peptide and creatinine. cTnI was measured using a chemiluminescent immunoassay (Abbott STAT High-Sensitivity Troponin I) on the Alinity i platform (Abbott Diagnostics, Abbott Park, IL, USA) at Drammen Hospital, Vestre Viken Hospital Trust. According to the manufacturer, cTnI has a limit of detection of 1.6 ng/L, a uniform URL of 26.0 ng/L and sex-specific URLs of 15.6 ng/L for women and 34.2 ng/L for men.^16^ cTnT was measured using the Elecsys Troponin T hs electrochemiluminescence immunoassay on a fully automated Cobas e 801 platform (Roche Diagnostics, Basel, Switzerland) at Akershus University Hospital. The 9-minute application was used for analysis. The manufacturer reports a limit of detection of 3 ng/L and a uniform URL of 14.0 ng/L. Sex-specific URLs are 9 ng/L for women and 16.8 ng/L for men.^17^

### Equity, diversity and inclusion statement

The author group includes both genders and comprises junior, mid-career and senior researchers from several fields, including cardiology, medical biochemistry and vascular physiology. The study cohort included male and female athletes of different ethnicities from various countries, although the majority were Caucasian.

### Data management and statistics

Data for cTnI, cTnT and cTnI/cTnT ratio were tested for normality with the Shapiro-Wilk test. Due to the markedly skewed data distribution, one-way repeated measures analysis of variance on ranks with time points as a within-subject factor was conducted for cTnI, cTnT and cTnI/cTnT ratio at all three time points. To correct for multiple comparisons, we used the Tukey test. Statistical significance was set to a two-tailed *p*-value of <0.05. Results are presented as mean±SD for continuous variables with normal distribution and as median (IQR) for continuous variables with considerably skewed distribution. All statistical analyses and plots were produced in SigmaPlot (V.15.0.0.13, Inpixon, Palo Alto, California, USA).

## Results

### Participants

**Figure 1:**
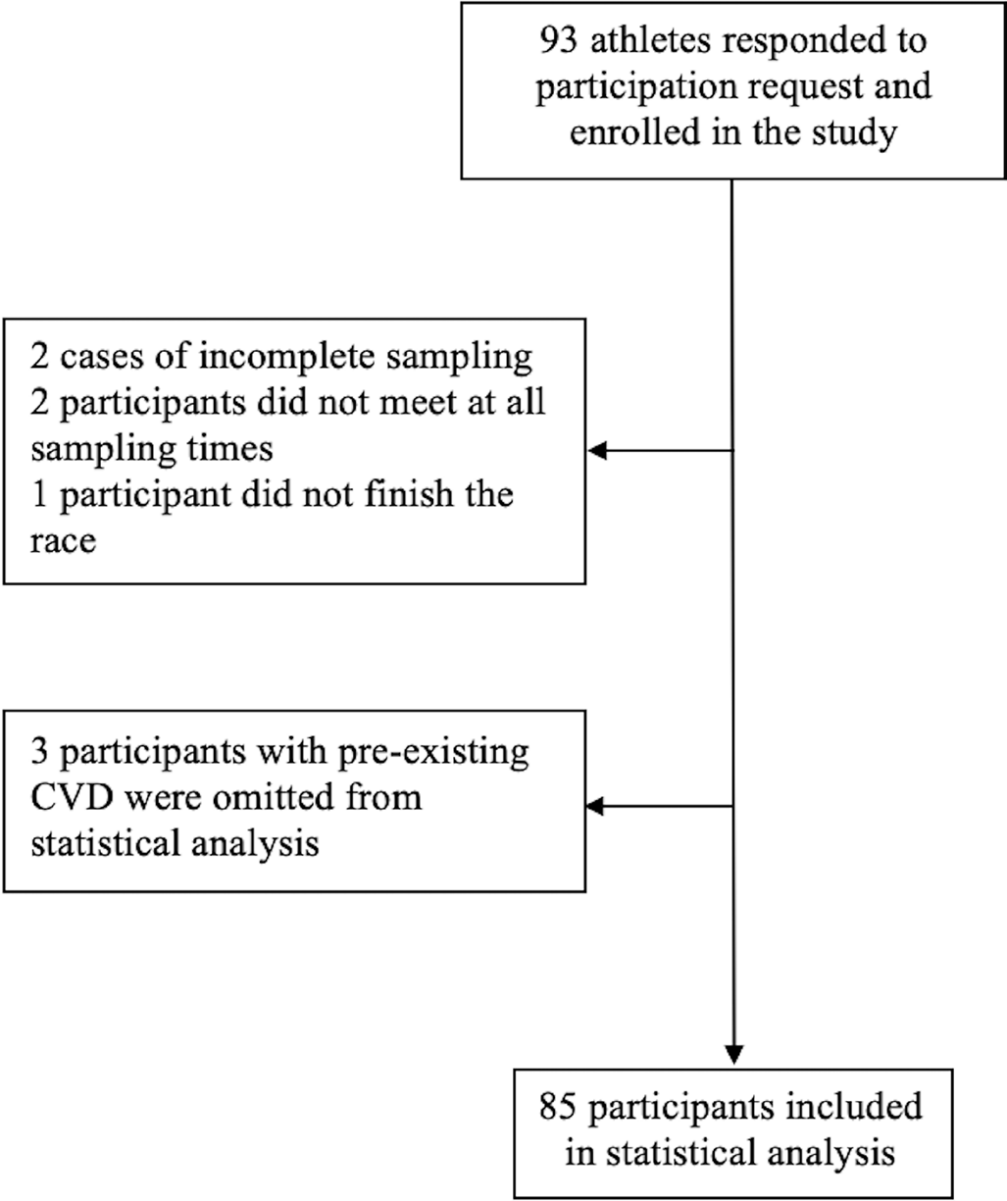
The inclusion of subjects for statistical analysis. CVD, cardiovascular disease.

A sample of volunteers from the race cohort was enrolled in the study. After excluding five subjects due to incomplete sampling and failure to complete the race (figure 1) and three subjects due to prior CVD, statistical analysis was performed on data from 22 women and 63 men. Baseline characteristics of the participants are presented in table 1. Among the three subjects excluded from statistical analysis due to pre-existing conditions, one had previously been diagnosed with obstructive CAD and had undergone coronary artery bypass graft surgery (CABG). Two athletes had pre-existing diagnoses of paroxysmal atrial fibrillation (AF). One subject with pre-existing AF reported three episodes of dyspnoea and sudden tachycardia during the race. The remaining participants reported no adverse events.

**Table 1:** Baseline characteristics in 85 participants included in the statistical analysis

Subject characteristics	Mean/median
Age, years	40.9±8.6
Females, n (%)	22 (25.9)
BMI, kg/m^2^	23.1±2.1
Body mass, kg	71.8±12.1
Total race time, hour	14.0±1.6
Swim time, hour:min	1:18 (1:12–1:30)
Bike time, hour:min	7:00 (6:36–7:36)
Run time, hour:min	5:18±0:48
Serum biomarkers	
NT-proBNP	
Proportion of respective group<35 ng/L, n (%)	54 (63.5)
Mean of participants>35, ng/L	55.0 (45.5–68.5)
Creatinine, μM	71.0±10.0
Haemoglobin, g/L	146±8
CRP, mg/L	1.0 (1.0–1.0)
Leucocytes, ×10^9^ /L	5.0±1.5
AST, U/L	26.7±7.6
Creatine kinase, U/L	161 (110–200)

Parametric data are given as mean values with standard deviation. Non-parametric data are given as median value with the 25th–75th percentile values.

AST, aspartate aminotransferase; BMI, body mass index; CRP, C-reactive protein; NT-proBNP, N-terminal pro-B-type natriuretic peptide.

### Timing of day after samples

Participants completed the race in a mean time of 14:00 hours:min (±1:36 hours:min), with race times ranging from 10:00 hours:min to 18:00 hours:min. The elapsed time from race-finish to the day-after sample ranged from 11:12 hours:min to 20:56 hours:min with a mean of 15:24 hours:min (± 1:56 hours:min). Individual sample times and cTn values the day after the race are illustrated in the supplementary files (online supplemental S2 figure 1).

### Cardiac troponin profiles

**Figure 2:**
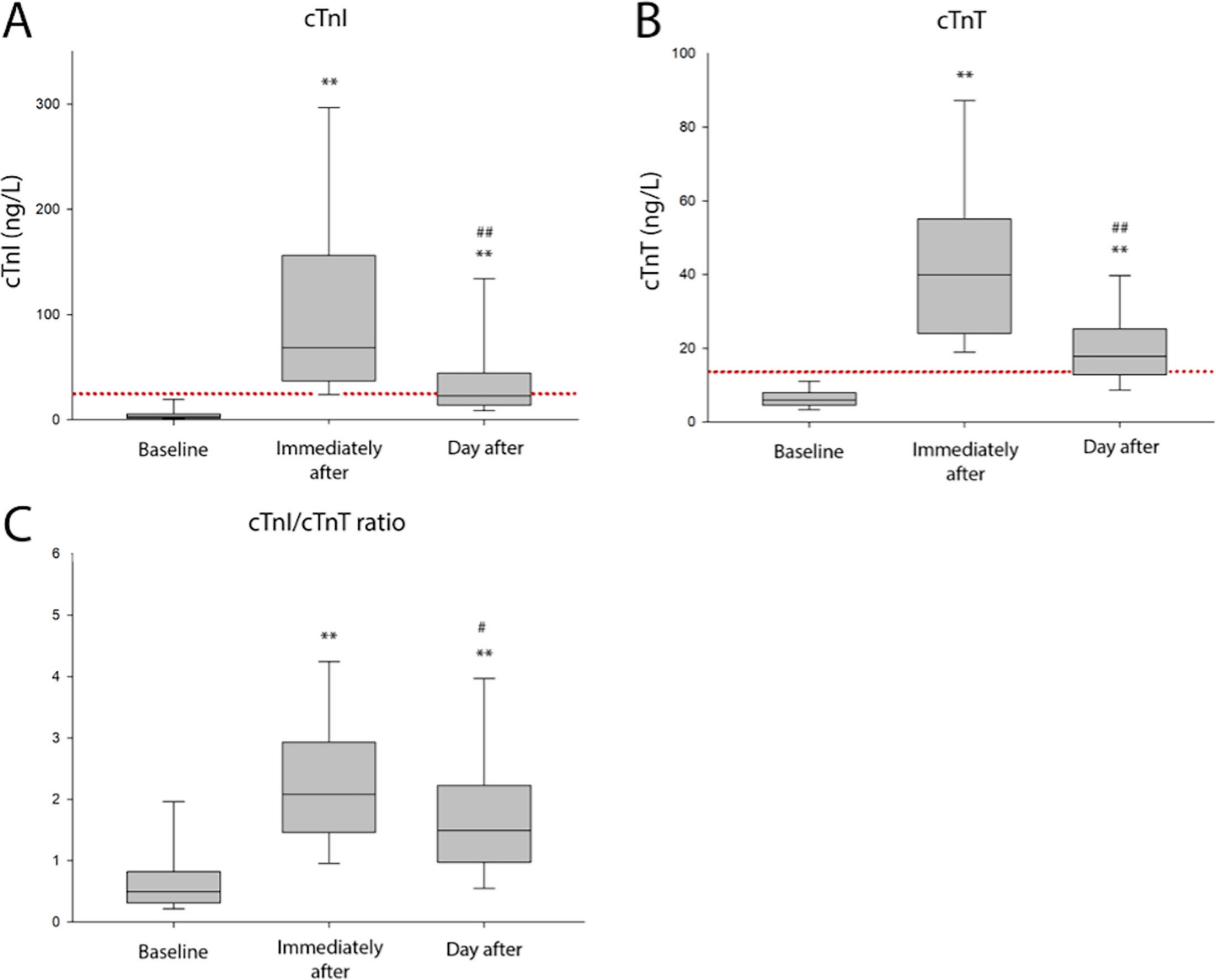
Box plots of cTnI, cTnT and cTnI/cTnT ratio at all sample times. In panels A, B and C, the box plot illustrates the median, 25th and 75th percentile and whiskers mark the 5th and 95th percentile. A double asterisk (**) marks a p-value of <0.001 compared with baseline. Hashes (# or ##) represent comparison with immediately after, with a single hash (#) marking a p-value of <0.05, and a double hash (##) marking a p-value of <0.001. The red dotted line demarks the combined URL for men and women for the applied cTnI and cTnT assays. cTnI, cardiac troponin I; cTnT, cardiac troponin T; URL, upper reference limit.

**Figure 3:**
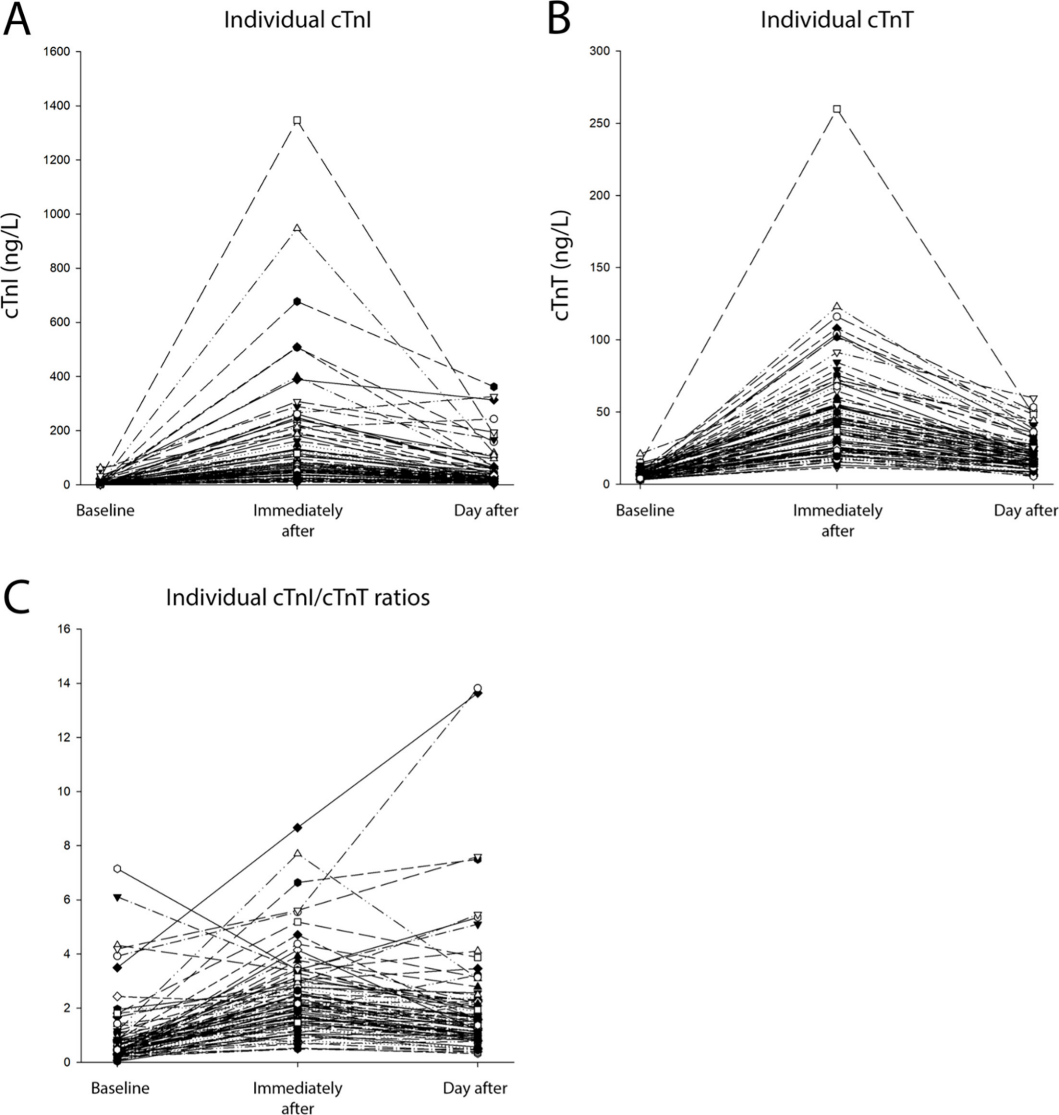
Line charts of individual results. Panel A displays cTnI concentrations, panel B shows cTnT concentrations and panel C illustrates cTnI/cTnT ratios at all sample times. cTnI, cardiac troponin I; cTnT, cardiac troponin T.

Median values, 25th and 75th percentiles for cTnI, cTnT and cTnI/cTnT ratio are presented in figure 2. Concentrations of cTnI rose markedly from baseline (3, (2–6) ng/L) to immediately after the race (69, (37–156) ng/L) and remained elevated compared with baseline the day after the race (23, (14–44) ng/L). Levels of cTnT increased from baseline (6, (5–8) ng/L) to immediately after (40, (24–55) ng/L) and remained higher than baseline the day after the race (18, (13–25) ng/L). The absolute reductions in median values for cTnI (47 ng/L) and cTnT (22 ng/L) from immediately after to the day after were statistically significant for both cTnI and cTnT. Concentrations of cTnI exceeded the sex-specific URL at baseline, immediately after finish, and the day after in 2%, 92% and 42% of participants, respectively. For the same time points, the proportions of subjects with cTnT above the sex-specific URL was 2%, 98% and 65%. The cTnI/cTnT ratio increased from baseline (0.5, (0.3–0.8)) to immediately after (2.1, (1.5–2.9)) and fell towards baseline the day after (1.5, (0.1–2.2)). Individual values for cTnI, cTnT and cTnI/cTnT ratio are presented in figure 3. Statistically significant changes to cTn concentrations remained after correcting for changes in haemoglobin concentration^18^ at both time points after the race. Haemoglobin-corrected cTn and creatinine concentrations are presented in the supplementary files (online supplemental S2 Table 1 and Table 2).

### Athletes with pre-existing cardiovascular disease

**Figure 4:**
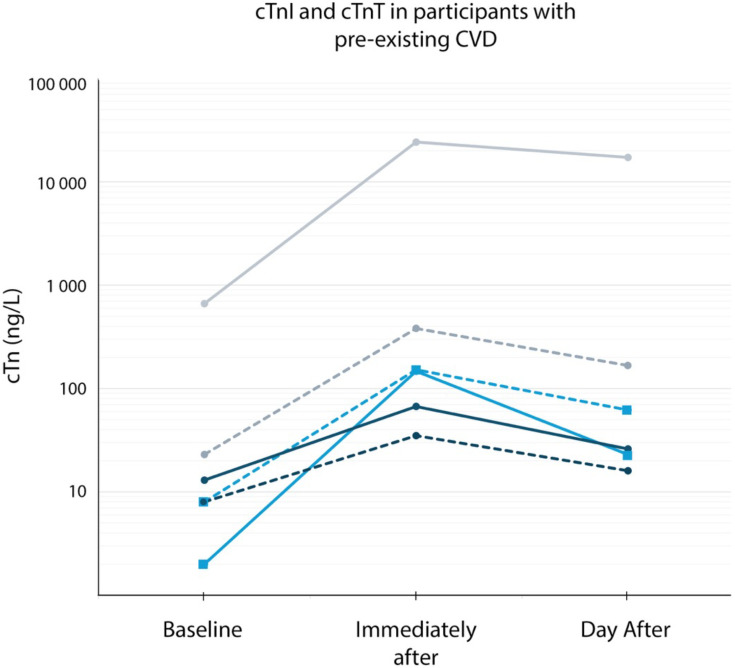
cTnI and cTnT in participants with pre-existing CVD. Full lines show cTnI concentrations, and dotted lines show cTnT concentrations. The grey lines represent participant 1 (CABG, asymptomatic). The light blue lines with squares represent participant 2 (AF, symptomatic). The dark blue lines represent participant 3 (AF, asymptomatic). AF, atrial fibrillation; CABG, coronary artery bypass graft surgery; cTnI, cardiac troponin I; cTnT, cardiac troponin T; CVD, cardiovascular disease.

Baseline characteristics of participants with pre-existing CVD are presented in the supplementary material (online supplemental S2 Table 3). The participant with CABG completed the race without any cardiovascular symptoms. His cTnI and cTnT peaked at 24 242 ng/L and 381 ng/L immediately after finish before declining the day after to 17 204 ng/L and 167 ng/L. His cTnI/cTnT ratio rose from 29 at baseline to 64 after finish and 103 the next day. Another participant with a history of AF reported three episodes of tachycardia accompanied by dyspnoea for an accumulated period of 35 min during the race. He described the present symptoms as similar to his past episode of AF. Symptoms resolved spontaneously on rest, and he completed the race. His cTn concentration was 8 ng/L at baseline, 152 ng/L after finish and 62 ng/L the day after the race. The cTnI/cTnT ratio was 0.3 at baseline, 1.0 after finish and 0.4 the day after. The second athlete with a history of AF reported no cardiovascular symptoms during the race. He displayed cTn values that closely followed the sample median at all time points. Individual results for cTnI and cTnT in athletes with pre-existing CVD are presented in figure 4.

## Discussion

### Main findings

The current study has two main findings. First, cTnI and cTnT increased markedly from baseline to immediately after the race. Accordingly, we observed concentrations above sex-specific URLs in 42% and 65% of participants the day after completing NXTRI. Second, the cTnI/cTnT ratio transiently increased immediately after the race, with most participants trending towards cTnI/cTnT ratio reduction the day after, see online supplemental figure 2.

### Cardiac troponins and endurance exercise

The high incidence of elevated cTn postexercise reported in our work is consistent with a recent study on triathletes as well as studies using high-sensitive cTn assays in endurance sports like cycling and running.^4 5 19 20^ Our findings should be interpreted in the light of the extreme and prolonged physical demands that Norseman triathlon requires. However, similar postexercise cTn elevations have been observed following sports events of shorter duration, including a 30-km running race, which was sufficient to exceed the URL of high-sensitivity cTnT and high-sensitivity cTnI, in 100% and 60% of the participants, respectively.^19^ In a larger study sample following a 91-km recreational mountain bike race, researchers observed increased cTnI and cTnT concentrations in all participants.^21^ The number with cTnI and cTnT concentrations exceeding the 99th percentile is unfortunately not provided in detail in that paper. However, the figures suggest that the great majority have postexercise concentrations >99th percentile for both cTnI and cTnT.^21^

The lower incidence rates of exercise-induced cTn elevation in earlier studies on triathletes^22^,^24^ may be partly due to differences in assay sensitivity and different reference population used to establish the URLs of older and newer cTn assays. Additionally, cTn levels have been linked to internal workload, power output, exercise duration and baseline blood pressure,^21 25 26^ which may vary between studies. Comparing cTn results between men and women in our cohort, differences in cTnI but not for cTnT or the cTnI/cTnT ratio, were significant between sexes at all time points (online supplemental S2 Table 4).

### Prognostic value and timing of postexercise cardiac troponin

A rise and fall in cTnT concentrations above the 99th percentile URL is an obligate criterion to diagnose myocardial injury.^27^ In our study, 83/85 participants without established CVD displayed cTnT concentrations exceeding the URL of 14.0 ng/L. However, in the absence of cardiac symptoms or ischaemic ECG changes this is likely a physiological response. The clinical relevance of elevated cTn after exercise in our cohort is unclear, but repeating blood samples might be helpful in determining which patients with exercise-induced cTn elevations require further diagnostic assessment or hospital care.

Elevated cTnI after long-distance walking is associated with increased risk of major adverse cardiovascular outcomes.^1^ Although the incidence of postrace cTnI concentrations exceeding the 99th percentile was low (9%) in this walking study, 27% of the participants with cTnI levels exceeding the URL compared with 7% of the participants with postexercise cTnI levels below the 99th percentile experienced either death or major adverse cardiovascular events during the follow-up period.^1^ However, other studies have observed no association between elevated cTn after exercise and mortality, coronary artery calcification, obstructive CAD and 6-year incidence of CAD.^2 3 5^ Taken together, there is conflicting evidence to support a prognostic value of cTn on mortality and a paucity of support for a prognostic value of cTn for other cardiovascular outcomes when cTn is sampled shortly after exercise.

Interestingly, Kleiven *et al* found an association between cTn elevation 24 hours after exercise and obstructive CAD,^5^ compatible with a prolonged release process. It is therefore possible that next-day cTn sampling is better suited for predicting subclinical CAD. The day after racing, 13% and 14% of NXTRI participants displayed cTnT and cTnI concentrations within the 95% CI of athletes with obstructive CAD in the cohort of Kleiven *et al* While we sampled cTn 15 hours postrace (online supplemental S2 figure 1) versus 24 hours in Kleiven *et al*, one could pose the question if prolonged cTn elevation may indicate that some NXTRI participants face a higher risk of subclinical obstructive CAD. However, such comparisons should be made with caution, given potential differences in cohort characteristics, race intensity and exercise modality.

### Characteristics of NXTRI cardiac troponin kinetics

Both intensity and exercise duration have been highlighted as important factors influencing exercise-induced cTn elevations.^26^ Furthermore, concentrations peak after 2–6 hour of exercise.^14^ In our study, we observed a negative linear association between cTnI, cTnT and I/T ratio and total race time. When adjusted for age and gender, these findings were no longer significant. This could be explained by the substantially longer exercise duration in our cohort, which exceeds the minimum time needed to reach peak cTn concentrations and explains why our values are at the declining phase. Additionally, the intensity level is typically lower during prolonged races compared with shorter sports events.

Overall, athletes with cTn values above the URL after triathlon finish, displayed falling cTn concentrations trending towards normalisation the following day. This aligns with reports of postexercise cTn returning to baseline within 24 hours to 72 hours after exercise.^6 14^ A recent human trial reports the elimination half-life for the Alinity hs-cTnI and Elecsys hs-cTnT assays as 213 min and 134 min, respectively.^28^ Our observations of day-after cTnI and cTnT concentrations of 33% and 45% of those sampled after finish may suggest continued cTn release in participants after the race finish. This interpretation agrees with past reports of exercise-induced peaks of cTn within 2 hours to 6 hours after exercise.^6 14^ Interestingly, three NXTRI participants displayed higher cTnI concentrations (377 ng/L, 265 ng/L and 49 ng/L) the day after than immediately after the race. One possible explanation for prolonged elevation of cTn is an ongoing release that exceeds the elimination rate and persists until the day after sample. A second possibility is that the present sampling within 1 hour of race finish missed a true cTnI peak within the beforementioned 2 hours to 6 hours postexercise window. If so, the apparent cTnI increases the day after could, in fact, be on a downward trajectory.

### Cardiac troponin I and T ratio

Our work demonstrates that the cTnI/cTnT ratio increases immediately after a long-distance triathlon race before trending towards baseline the day after the race. This group response differed from the considerably higher and increasing cTnI/cTnT ratio seen in a single participant with pre-existing CAD and CABG. These observations should be considered in the context of prior studies reporting on cTnI/cTnT ratios. In patients diagnosed with MI after presenting to the emergency department with acute chest pain, the cTnI/cTnT ratio is shown to discriminate between type 1 MI and type 2 MI.^9^ Median cTnI/cTnT ratios in hospitalised patients with MI range from 1.2 (type 2 MI) and 3.5 (type 1 MI) 2 hour after presentation to 11 (MI) 23 hours after presentation.^9 10^ Although not explicitly demonstrated, this limited body of evidence could imply that the cTnI/cTnT ratio may be expected to increase during the initial 24 hours of myocardial injury.

While the mechanisms underlying elevated cTnI/cTnT ratios during myocardial injury are poorly characterised, animal models and in vitro experiments suggest cTnI is cleaved and released at a faster rate than cTnT. While the lack of postrace clinical examinations in our work prevents strict conclusions to be drawn, we believe the falling cTnI/cTnT ratio the day after exercise speaks against the presence of severe necrotic injury at group level. That said, a few interesting observations should be highlighted. Fourteen NXTRI participants showed increasing cTnI/cTnT ratio between finish and the day after, all with cTn above the URL the day after. The distribution of cTnI/cTnT ratios at each sampling point is presented in the supplementary material (online supplemental S2 figure 2). Furthermore, the NXTRI participant with a history of CABG showed an outlier cTnI/cTnT ratio at baseline (29), immediately after the race (64) and the day after the race (103). The high peaks of cTnI (24 242 ng/L) and cTnT (381 ng/L) could raise the suspicion of ischaemic myocardial injury. Interestingly, he completed the race with no reports of cardiovascular symptoms and at 6-year follow-up, he denied signs or symptoms of acute cardiac events since his NXTRI participation.

### Strengths and limitations

This study observed participants during exposure to physical workloads and ambient environments outside of what is typically feasible in a laboratory setting. The omission of pre- and postrace clinical examinations, including electrocardiograms, is an obvious limitation that was deemed necessary due to the challenging logistics of this race. Therefore, we cannot entirely rule out a confounding effect of pre-existing undetected disease and subclinical cardiac events. For this reason, participants with cTn values in the upper quartile at any sample point were contacted in December 2024 and questioned about any cardiovascular events and diagnostics in the period following their race. This effort yielded an 82.8% response rate, of which no athletes reported any cardiovascular events or had been diagnosed with CVD since their participation.

Furthermore, baseline blood pressure, power output and time accumulated at high absolute heart rates are associated with postexercise cTn release.^21 25 26^ Taking these variables into account would have strengthened our results. Logistics only allowed sampling during a brief time window the day after the race. This resulted in a variance in the elapsed time from sampling at the race finish to sampling the day after (online supplemental S2 figure 1). In addition, we acknowledge that different cTnI assays may provide differing results. Lastly, our data are descriptive and do not provide any new information concerning mechanisms for cTn release, nor does it address whether cTn release is due to transient or permanent cardiomyocyte damage. We believe future studies would benefit from a third postrace sampling point and that anchoring sampling to the time since exposure would better elucidate the kinetics of postexercise cTn release.

## Conclusion

This study shows that cTnI, cTnT and the cTnI/cTnT ratio are increased immediately after a long-distance triathlon race and trend towards normalisation the next day. We also observed a prolonged cTn elevation in a substantial proportion of participants and increasing cTnI/cTnT ratios in individual participants the day after the race. The potential clinical implications of these observations remain unknown. Future longitudinal studies should seek to clarify the potential prognostic value of elevated cTn and cTnI/cTnT ratios at several sampling points after triathlon and endurance races in general.

## Supplementary material

10.1136/bmjsem-2025-002810 online supplemental file 1

10.1136/bmjsem-2025-002810 online supplemental file 2

## Data Availability

All data relevant to the study are included in the article or uploaded as supplementary information.
