# Supplementary material for: Cardiac troponin patterns in athletes completing Norseman Xtreme Triathlon
Source: BMJ Open Sport Exerc Med. 2026 May 17;12(2):e002810. doi: 10.1136/bmjsem-2025-002810 (PMC13182439; doi:10.1136/bmjsem-2025-002810)
Supplement: online supplemental file 2 [file bmjsem-12-2-s002.pdf]

S2 - Supplementary text, tables and figures

Selection of subjects for statistical and descriptive analysis

To eliminate obvious confounders, participants with established cardiovascular disease (CVD), including CAD, hypertension, atherosclerosis, arrhythmias, or medical history of cardiac surgery, were not included in the main statistical analysis. However, descriptive data from these participants are reported. This was also the case for athletes with other conditions that could impact cardiovascular function, such as renal disorders or diabetes mellitus. The inclusion process is illustrated in Figure 1.

This document contains supplementary tables and figures to the manuscript Increased High-Sensitivity Cardiac Troponin and High-Sensitivity Cardiac Troponin I to T Ratio in Triathletes Following Norseman Xtreme Triathlon.

The following tables and figures are included:

Supplementary table 1: **Hb-corrected cTn** – page 2

Supplementary table 2: **Creatinine concentrations** – page 2

Supplementary figure 1: **Timing of individual cTn sampling** – page 3

Supplementary table 3: **Baseline characteristics of athletes with CVD** – page 4

Supplementary table 4: **Sex-specific cTn results** – page 5

Supplementary figure 2: **cTnI/cTnT ratio distributi**on – page 6

Parameter	Baseline	Immediately after	Day after
Hb-corrected cTnI, ng/L	3.4 [1.7-6.0]	70.7 [38.1-161.8], *p* <0.001	24.6 [15.2-46.3], *p* <0.001
Hb-corrected cTnT, ng/L	6.0 [4.6-8.1]	41.3 [25.1-58.4], *p* <0.001	19.3 [13.7-26.7], *p* <0.001
Hb-corrected cTnI/cTnT ratio	0.5 [0.3-0.8]	2.1 [1.5-2.9], *p* <0.001	1.5 [1.0-2.2], *p* =0.02

Supplementary Table 1. Hemoglobin-corrected concentrations for cTnI, cTnT, and cTnI/cTnT ratio. *P*-values given immediately after are compared to baseline values. *P*-values given the day after are compared to corrected values immediately after. Hb: Hemoglobin.

Parameter	Baseline	Immediately after	Day after
Creatinine, µmol/L	71.0 ± 9.7	88.6 ± 16.6, *p* <0.001	81.4 ± 16.9, p<0.001

Supplementary Table 2. Creatinine concentrations at baseline, immediately after, and the day after. *P*-values are compared to baseline concentrations.

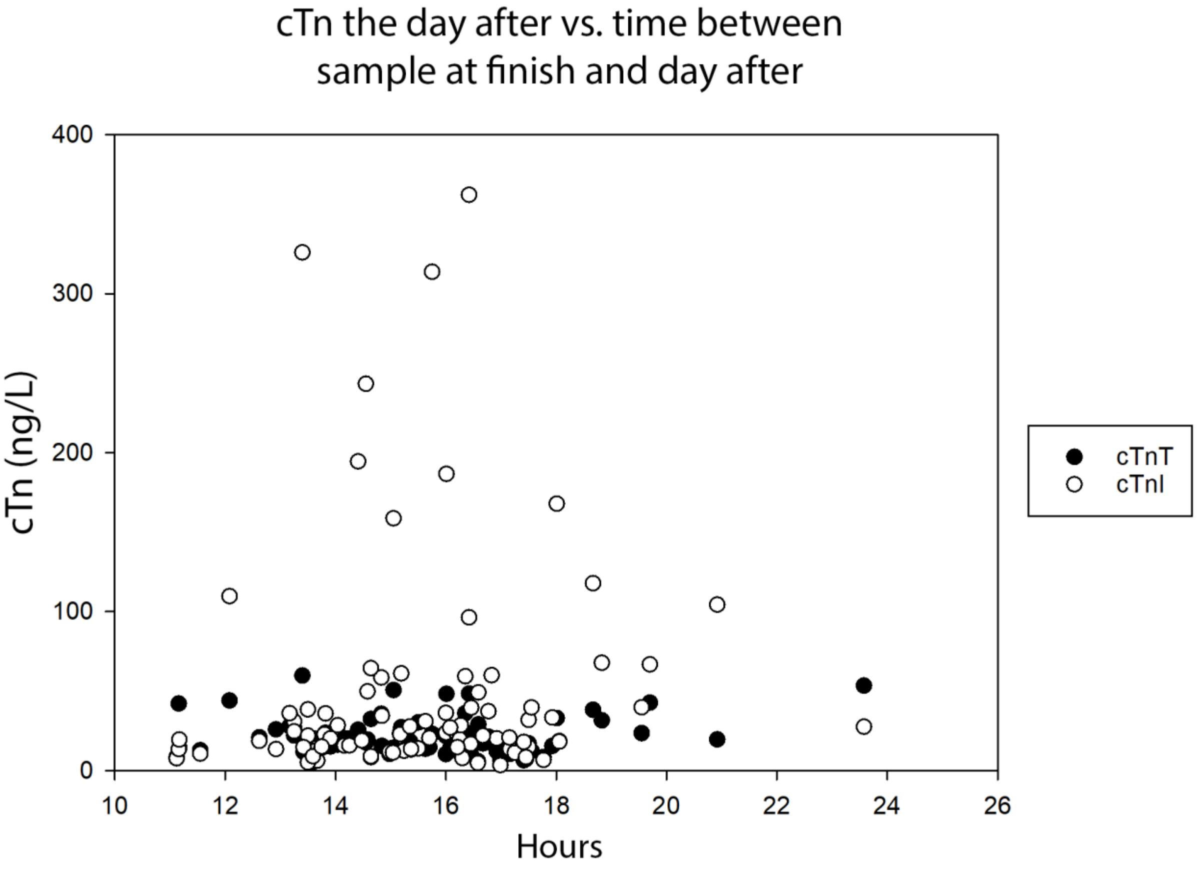

Supplementary Figure 1. Hours elapsed between sampling immediately after finish and the day after versus individual cTnI and cTnT the day after the race. White dots represent cTnI and black dots represent cTnT. cTnI: Cardiac troponin I; cTn T: Cardiac troponin T.

Baseline characteristics	Athlete 1 (CABG)	Athlete 2 (AF)	Athlete 3 (AF)
Age, years	61	35	39
NT-ProBNP, ng/L	75	49	<35
Creatinine, µM	78	58	82
Hemoglobin, g/L	176	143	150
CRP, mg/L	1	1	1
Leukocytes, x 10^9/L	6,1	6	6
AST, U/L	24	30	28
Creatinine Kinase, U/L	119	304	132
**Cardiac troponin results**			
cTnI baseline, ng/L	663	2	13
cTnI after finish, ng/L	24 242	147	67
cTnI day after, ng/L	17 204	23	26
cTnT baseline, ng/L	23	8	8
cTnT after finish, ng/L	381	152	35
cTnT day after, ng/L	167	62	16
cTnI/cTnT baseline	29	0.3	1.6
cTnI/cTnT after finish	64	1.0	1.9
cTnI/cTnT day after	103	0.4	1.7

Supplementary Table 3. Baseline characteristics and individual cTn and cTnI/cTnT ratio results for the three participants with pre-existing cardiovascular disease. Athlete 1 was the participant with pre-existing coronary artery disease and a history of coronary bypass graft surgery. Athlete 2 was the athlete with a history of atrial fibrillation who experienced symptoms of likely AF during the NXTRI. Athlete 3 had a history of atrial fibrillation. Athletes 1 and 3 completed the NXTRI without cardiovascular symptoms. NT-proBNP: N-terminal pro-B-type natriuretic peptide; CRP: C-reactive protein; AST: Aspartate Aminotransferase.

Characteristic	Women (n=22)	Men (n=63)	*p*-value
cTnI			
Baseline, ng/L	2.1 [1.2 - 4.9]	3.5 [1.9 - 6.9]	0.01
Immediately after, ng/L	53.0 [27.3 - 82.8]	75.3 [44.5 - 212.1]	0.05
Day after, ng/L	14.7 [10.1 - 29.0]	27.5 [15.7 - 59.9]	0.01
cTnT			
Baseline, ng/L	4.5 [3.3 - 7.1]	6.3 [5.2 - 8.4]	0.001
Immediately after, ng/L	37.6 [18.4 - 48.5]	41.7 [25.1 - 60.5]	0.10
Day after, ng/L	14.1 [8.7 - 23.9]	19.5 [14.2 - 25.8]	0.04
cTnI/cTnT ratio			
Baseline, ratio	0.4 [0.3 - 0.9]	0.6 [0.3 - 0.9]	0.14
Immediately after, ratio	1.7 [1.3 - 2.2]	2.2 [1.5 - 3.0]	0.07
Day after, ratio	1.2 [0.8 - 2.2]	1.6 [1.0 - 2.2]	0.15

Supplementary table 4. Sex-specific results for high-sensititivy cardiac troponin I, high-sensitivity cardiac troponin I and cardiac troponin I/cardiac troponin T ratios. The p-value refers to comparison between men and women with the Mann-Whitney Rank Sum test.

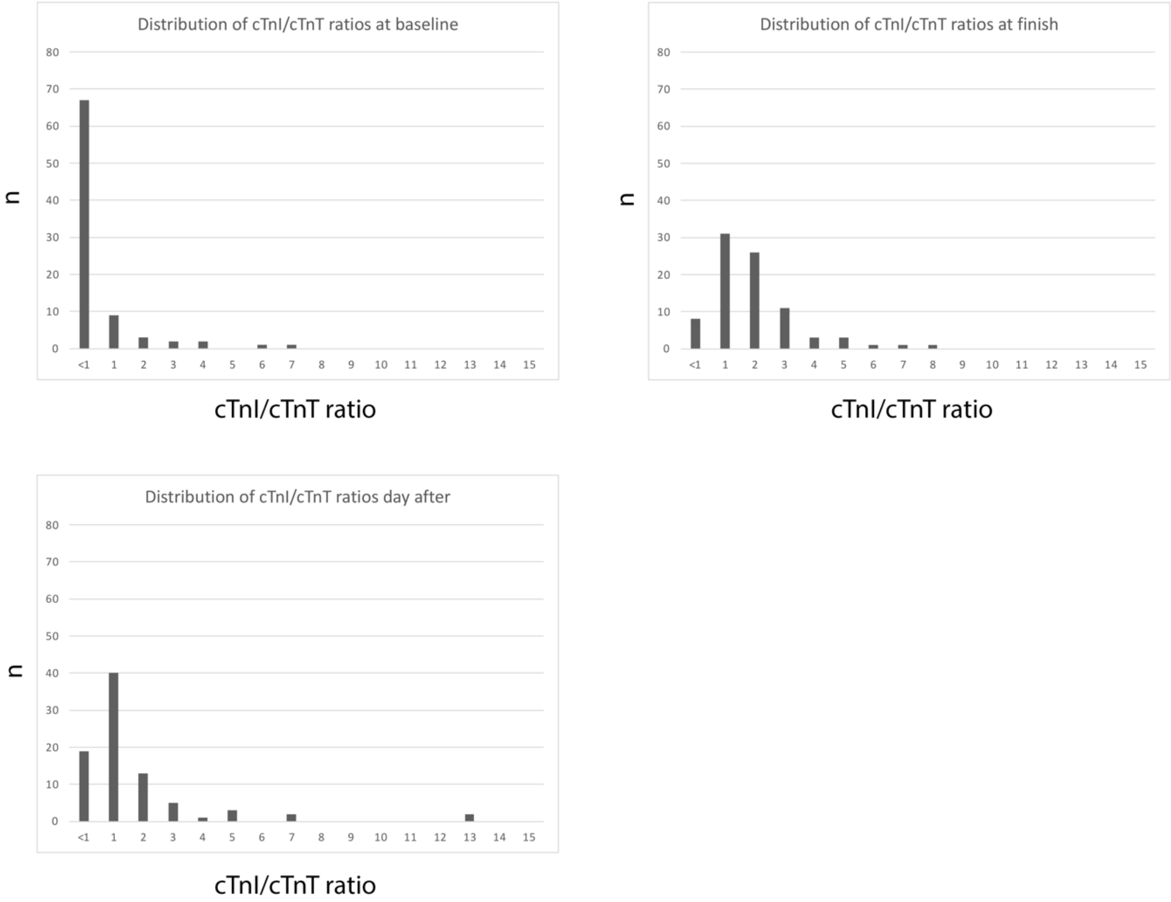

Supplementary Figure 2. Distribution of cTnI/cTnT ratios at baseline, immediately after the race, and the day after the race. Participants with pre-existing CVD are not shown. The value 1 on the x-axis represents a ratio from 1.0 – 1.99, the value 2 represents 2.0 – 2.99 and so on. The y-axis represents the number of participants.
